# rTMS Reduces Psychopathological Burden and Cocaine Consumption in Treatment-Seeking Subjects With Cocaine Use Disorder: An Open Label, Feasibility Study

**DOI:** 10.3389/fpsyt.2019.00621

**Published:** 2019-09-05

**Authors:** Mauro Pettorruso, Giovanni Martinotti, Rita Santacroce, Chiara Montemitro, Fabrizio Fanella, Massimo di Giannantonio, the rTMS stimulation group, Luisa De Risio

**Affiliations:** ^1^Department of Neuroscience, Imaging and Clinical Sciences, G.d’Annunzio University, Chieti, Italy; ^2^La Promessa o.n.l.u.s., Rome, Italy; ^3^Department of Pharmacy, Pharmacology and Postgraduate Medicine, University of Hertfordshire, Hatfield, United Kingdom

**Keywords:** transcranial magnetic stimulation, cocaine use disorder, addiction, anhedonia, anxiety, depression

## Abstract

**Introduction:** Cocaine use disorder (CUD) currently represents a notable public health concern, linked with significant disability, high chances of chronicity, and lack of effective pharmacological or psychological treatments. Repetitive transcranial magnetic stimulation (rTMS) is supposed to be a potential therapeutic option for addictive disorders. Aim of this study was to evaluate the feasibility of rTMS on (1) cocaine craving and consumption and (2) other comorbid psychiatric symptoms.

**Methods:** Twenty treatment seeking CUD subjects underwent 2 weeks of intensive rTMS treatment (15Hz; 5 days/week, twice a day for a total of 20 stimulation sessions) of the left dorsolateral prefrontal cortex, followed by 2 weeks of maintenance treatment (15Hz, 1 day/week, twice a day). Sixteen patients completed the study. Patients were evaluated at baseline (T0), after 2 weeks of treatment (T1), and at the end of the study (T2; 4 weeks), with the following scales: Cocaine Selective Severity Assessment (CSSA), Zung Self-Rating Anxiety Scale, Beck Depression Inventory (BDI), Symptom Checklist-90 (SCL-90), and the Insomnia Severity Index.

**Results:** After four weeks of rTMS treatment, 9 out of 16 subjects (56.25%) had a negative urinalysis test, with a significant conversion rate with respect to baseline (Z = −3.00; p = 0.003). Craving scores significantly improved only at T2 (p = 0.020). The overall psychopathological burden, as measured by the SCL-90 Global Severity Index (GSI), significantly decreased during the study period (Z = −2.689; p = 0.007), with a relevant improvement with regards to depressive symptoms, anhedonia, and anxiety. Subjects exhibiting lower baseline scores on the SCL-90 were more likely to be in the positive outcome group at the end of the study (Z = −3.334; p = 0.001).

**Discussion:** Findings from this study are consistent with previous contributions on rTMS use in subjects with cocaine use disorder. We evidenced a specific action on some psychopathological areas and a consequent indirect effect in terms of relapse prevention and craving reduction. A double-blind, sham-controlled, neuro-navigated rTMS study design is needed, in order to confirm the potential benefits of this technique, opening new scenarios in substance use disorders treatment.

## Introduction

Cocaine use disorder (CUD) currently represents a notable public health concern, linked with significant disability, high chances of chronicity, and considerable mortality (1). In Italy, it has been recently estimated that up to 4.8% of subjects aged 15–64 consumed cocaine at least once in their lifetime, whereas 1.3% has been diagnosed with CUD (2). Unfortunately, so far there is still uncertainty with regards to the actual effectiveness of pharmacological or psychological treatments proposed for CUD. In the past few years, both preclinical and human neuroimaging studies evidenced a relationship between altered brain functions and behaviors observed in addicted patients, such as lack of impulse control, drug-seeking compulsions, and inability to modulate behaviors according to the different circumstances. More specifically, cocaine consumption, especially if long-term, has been associated with structural (e.g., brain volume reduction) (3, 4) and functional [e.g., reduced cortical activity (5–7), impaired executive functions, and dysregulated neurotransmission (8–10)]. Moreover, preclinical researches highlighted that a dysregulated inhibitory control, which may be due to impaired prefrontal cortex (PFC) functions, has a key role in compulsive drug-seeking behaviors, increasing drug intake and addiction severity (11).

Neuromodulation interventions have greatly developed in light of these new insights. In fact, they may offer researchers and clinicians the possibility not only to study altered brain pathways, but also to act on them, directly focusing on affected circuits needing to be reshaped. Transcranial magnetic stimulation (TMS) is a noninvasive technique able to induce an electric flow in targeted brain regions (12). TMS pulses may be delivered in sequences: this can determine long‐term changes, modifying cellular excitability and resulting behaviors. The promoting or suppressing effect depends on a number of parameters, such as stimulation site and type of sequence. In psychiatry, most of the research involving TMS has been performed on mood disorders, and in particular Major Depressive Disorder, while few studies investigated the potential application of TMS in manic episodes (13). rTMS has also been applied as a therapeutic option in schizophrenia (14), obsessive–compulsive disorder (15), and impulsive-compulsive disorders (16, 17). rTMS is still at a very early stage of study in the field of addiction; it has been mostly investigated for its potential anti-craving action. So far, most studies on human samples have dealt with nicotine, stimulants and alcohol use disorders, targeting left or right dorsolateral prefrontal cortex (DLPFC) (18–20). Notable limitations of these studies are that the vast majority only included one or two stimulation sessions, the sample sizes were rather limited, and frequently there was no control group. To date, the largest clinical trial in the addiction field revealed that after 13 sessions of 10 Hz stimulation, a group of cigarette smokers significantly reduced their self-reported smoking habit, and their 6-month abstinence rates were better when compared to sham group (21). With regards to stimulants use disorders, studies on animal models, applying ontogenetic stimulation to the medial prefrontal cortex (mPFC) of cocaine-addicted rats, demonstrated it was able to prevent compulsive cocaine seeking behaviors (22). The therapeutic effect may be due to a combination of variables, including a modulation on the activity of the reward circuit via the glutamatergic PFC efferents. High frequency rTMS of the right DLPFC has been able to reduce craving in a sample of cocaine addicted patients (this was not true for rTMS of the left DLPFC) (23). A study by Politi and colleagues, published in 2008, highlighted a notable self-reported reduction in craving among thirty-six cocaine users who underwent 10 sessions of 15 Hz TMS on the left DLPFC (600 pulses, 100% rMT) (24). There was, however, no active sham control in this study. Another study investigating the effects of rTMS on the DLPFC in cocaine users included thirty-two individuals with cocaine use disorder, who were randomized to receive either rTMS (eight sessions, 15 Hz DLPFC TMS, 2,400 pulses, 100% rMT, daily for the first 5 d, once a week for the following 3 weeks) or a pharmacological treatment. After 29 d, the rTMS group reported significantly less craving and was significantly more abstinent than the pharmacotherapy group. Other studies confirmed these preliminary data, suggesting that rTMS of the PFC may determine a reduction in cocaine consumption and minimize relapse risk (25–27).

Aim of our research is 1) to evaluate the effectiveness of rTMS targeting the left DLPFC on cocaine craving and consumption; 2) to evaluate its effect on other psychiatric variables (general symptomatology, hedonic state, mood, anxiety, insomnia, suicidality).

## Materials and Methods

Twenty treatment-seeking patients, aged 18–65 and meeting the diagnostic criteria for Cocaine Use Disorder (CUD) according to DSM-5, were enrolled in the study. All participants were physically healthy, and had no other current major Axis I diagnosis (schizophrenia spectrum disorders, bipolar I disorder, post-traumatic stress disorder), including current abuse or dependence to other substances (with the exception of nicotine).

The patients were selected according to the following criteria:

- aged 18–65;
- diagnosed with a moderate to severe Cocaine Use Disorder according to DSM-5 criteria;
- no comorbid diagnosis of other Substance Use Disorders (besides nicotine), Bipolar Disorder type 1, Schizophrenia, or other psychotic disorders;
- no history of seizures or other relevant neurological disorders, including organic brain disease, epilepsy, stroke, brain lesions, multiple sclerosis, previous neurosurgery, or personal history of head trauma that resulted in loss of consciousness for >5 min and retrograde amnesia for >30 min;
- ferromagnetic, or other magnetic-sensitive metal implants;
- no current use of pro-convulsant drugs;
- for female patients: no pregnancy/breastfeeding.

The study was approved by the local Ethical Committee and all participants were informed about study procedures and provided written informed consent before the beginning of the experiment, in line with the Helsinki Declaration.

## Study Procedures

The study consisted of: 1) an outpatient screening phase, during which patients were screened to assess their eligibility to be enrolled in the study, 2) an intensive rTMS treatment phase, during which the subjects received 20 stimulation sessions (2 daily, 5 d/week) for 2 weeks, 3) a maintenance phase of 2 weeks, during which the subjects received two consecutive rTMS sessions once a week.

## rTMS Device and Protocol

Repetitive TMS was delivered using a MagPro R30 with the Cool-B80 figure-of-eight coil (MagVenture, Falun, Denmark), allowing for a focal stimulation of the DLPFC.

Every session began with the determination of resting motor threshold (RMT), used to calculate the intensity of stimulation. Subjects were seated in a recliner with their hands in a comfortable resting position, wearing earplugs and a cap over the scalp, and electrodes were taped over the region of the abductor pollicis brevis (APB) belly and associated tendon of the right hand. The RMT was considered as the minimum single-pulse stimulator output intensity resulting in motor evoked potentials (MEPs) of the abductor pollicis brevis (APB) of at least 50 μV peak-to-peak amplitude in ≥50% of pursued trials (≥5/10; Rossini-Rothwell method) (28).

To localize DLPFC, we used the BeamF3 method (29) a system to find the F3 position using three skull measurements. By using the circumference and distances between skull anatomical landmarks, it locates DLPFC with a reasonable approximation to MRI-guided neuronavigation system (30). This coil location was marked on the cap, in order to ensure accuracy and consistency across sessions.

Two consecutive rTMS sessions lasting 13 min each were performed, with a minimum of 60 min interval between sessions. Each rTMS session was delivered at the intensity of 100% of the individual resting motor threshold, for a total of 40 trains (60 stimuli per train, inter-train interval of 15 s, for a total of 2,400 stimuli). At the beginning of each session, participants were exposed to cocaine-related cues for approximately 2 min and instructed to focus and inhibit any craving elicited by the cues. These procedures were proposed in reason of several evidences suggesting the use of a cue-exposure paradigm to engage targeted circuits and to improve stimulation outcomes (31). In fact, cue-induced craving is linked to impaired activity in prefrontal areas and fronto-striatal circuits. Eliciting cocaine-related cues in cocaine addicts was supposed to elicit activation of the executive control network and to increase the ability to modulate craving in a circuit-specific manner through non-invasive brain stimulation techniques (32). Also, at the end of the session, the ‘Side Effect” questionnaire was administered to evaluate potential side effects.

The Ethic Committee of the University “G.d’Annunzio” of Chieti-Pescara approved this study. All subjects signed a written informed consent according to the Declaration of Helsinki.

## Psychometric Assessment

Clinical and psychometric data were acquired at baseline (T0), after two (T1) and four weeks (T2) of rTMS treatment. The psychometric assessment included self-administered and physician-administered tests, chosen to evaluate cocaine-related withdrawal symptoms and psychopathologic symptoms.

All the patients underwent the following psychometric evaluation:

- the Cocaine Selective Severity Assessment (CSSA), a clinician-administered scale aimed at evaluating cocaine withdrawal signs and symptoms; it includes items exploring craving, hedonic tone, suicidal ideation, appetite, irritability, energy;
- the Beck Depression Inventory, to assess depressive symptoms, such as hopelessness, irritability and guilt, as well as physical symptoms such as fatigue, weight loss, and diminished interest in sexual activities;
- the Zung Self-Rating Anxiety Scale, to assess anxiety levels in terms of cognitive, autonomic, motor and central nervous system symptoms;
- global psychopathology was explored by using the Symptom Checklist-90 (SCL-90), a self-report psychometric instrument used to measure broad range of psychopathological distress into nine symptomatic dimensions;
- the Insomnia Severity Index insomnia (ISI), a seven-item questionnaire evaluating the severity of nighttime and daytime components of sleep disorders.

## Statistical Analysis

Statistical analysis was performed using SPSS for Windows, Version 20.0 (SPSS Inc, Chicago, Illinois). All analyses were conducted using non-parametric testing. Descriptive statistical analyses were provided at baseline for categorical (number and percentage) and continuous (mean, standard deviation, range min-max) data. Wilcoxon Test for paired variables was used to monitor changes in scores on psychometric scales between baseline and follow-up measures. Mann-Whitney U test was used to investigate overall psychopathological differences between subjects reaching or not a positive outcome at the end of the study. The significance threshold was set at 0.05.

## Results

Twenty patients were enrolled, sixteen (80%) completing all the follow-up visits during the study period, while four had an early drop-out and were excluded from the trial. The treated subjects reported no significant side effect. Fourteen out of sixteen patients were male (87.5%), mean age of the sample was 36.63 years old (SD: 6.29, range 27–51). Mean educational level was 12.12 years (SD: 2.87, range 8–18); most of the subjects (62.5%) were employed during their participation to the study. Subjects had a mean duration of cocaine addiction of 15.37 years (SD: 5.58, range 8–27); half of the sample had an ongoing pharmacological treatment (33). Subjects did not report any specific psychotherapeutic regimen in the month before the recruitment in the study. Other demographic and clinical data are reported in **Table 1**.

**Table 1:** Demographic and clinical characteristics at baseline (M ± SD and ranges).

	Mean ± SD (range)
N.	16
Gender (M/F; %M)	14/2 (87, 5%)
Age	36.63 ± 6.29 (27–51)
Education	12.12 ± 2.87 (8–18)
Employed (Yes; %)	10 (62.5%)
Marital Status	
* Married*	6 (38%)
* Separated/divorced*	2 (13%)
* Single*	8 (50%)
Duration of cocaine addiction	15.37 ± 5.58 (8–27)
Form of cocaine assumed (Powder/Crack)	14/2
Nicotine Use (n.; %)	10 (62.5%)
Addiction comorbidity (n.; %)	3 (18.8%)
Cannabis Use	0 (0%)
Alcohol Use	3 (19%)
Benzodiazepine Abuse	1 (6%)
Audit	6 ± 5.02
Current pharmacological treatment (n.; %)	8 (50%)
Antidepressants	3 (18.7%)
Mood stabilizers	7 (43.7%)

## Cocaine Use and Craving

At the baseline, all patients declared cocaine use in the previous week, and all had a positive urinalysis test for cocaine. After four weeks of rTMS treatment, 9 out of 16 subjects (56.25%) had a negative urinalysis test, with a significant conversion rate with respect to baseline (Z = −3.00; p = 0.003). With regards to cocaine-related withdrawal symptoms, as measured by the CSSA scale, mean total score at the baseline (T0) was 36.07 (SD 18.39). Patients were re-evaluated after two (T1) and four (T2) weeks of rTMS treatment: CSSA mean total score showed a consisted and statistically significant decrease (T1: 17.43; T2: 16:44—ΔT1-T0: p = 0.002; ΔT2-T0: p = 0.008). Craving, described as the urge or intense desire to consume cocaine, evaluated by the CSSA craving subscale, highlighted an after-treatment significant improvement from baseline to T2 (T0 mean score: 4.43; T2 mean score: 2.93 — ΔT2-T0: p = 0.02), but not from baseline to T1.

## Psychiatric Symptoms

The overall psychopathological burden, as measured by the SCL-90 Global Severity Index (GSI), significantly decreased within the study period (Z = −2.689; p = 0.007).

Comorbid depressive symptoms in the sample were assessed by the BDI and the SCL-90 Depression subscale: both confirmed a significant reduction from T0 to T2. BDI mean score at T0 was 17.19, suggestive of mild depression; mean scores at T1 (7.25) and T2 (7.38) indicated no signs of depression (ΔT1-T0: p = 0.003; ΔT2-T0: p = 0.008). Similarly, SCL-90 Depression subscale had a mean score of 1.24 at T0, which decreased to 0.54 and 0.67 at T1 and T2, respectively (ΔT1-T0: p = 0.004; ΔT2-T0: p = 0.003).

Anhedonia, described as the reduced ability to feel pleasure for natural rewards, was assessed by means of a CSSA subscale, and a statistically significant improvement of this symptom was evidenced at both T1 and T2 (ΔT1-T0: p = 0.027; ΔT2-T0: p = 0.033).

Suicidal ideation, evaluated by the CSSA subscale, did not reach an after-treatment significant improvement (ΔT1-T0: p = 0.059). However, mean score was extremely lower since the beginning of the study (see **Table 2**).

**Table 2:** Cocaine-related withdrawal symptoms as measured by Cocaine Selective Symptoms Assessment (CSSA) at the baseline and after two weeks (T1) and four weeks (T2) of rTMS treatment. Relevant subscales have been reported.

	T0	T1	T2	% ΔT1-T0	% ΔT2-T0	Sig. ΔT1-T0	Sig. ΔT2-T0
**CSSA total score**	36.07 ± 18.39	17.43 ± 10.55	16.44 ± 8.48	–51.7%	–54.4%	**.002**	**.008**
Craving	4.43 ± 3.63	3.29 ± 3.26	2.93 ± 3.24	–25.7%	–33.9%	.342	**.020**
Anhedonia	2.69 ± 2.92	0.60 ± 1.24	0.69 ± 1.31	–77.7%	–74.3%	**.027**	**.033**
Suicidal ideation	0.93 ±1.54	0 ± 0	0.31 ± 1.1	–100.0%	–66.7%	.059	.083

Bolded text: p < 0.05

Anxiety, assessed by means of the Zung Self-Rating Anxiety Scale (SAS), was apparently not a primary symptom in the selected sample: the mean score (36.37) at T0 is considered to be in the normal range. However, it showed a steady decrease after treatment (T1: 33.2; T2: 28 — ΔT2-T0: p = 0.001). On the other hand, considering the Anxiety subscale of the SCL-90, the mean score registered at T0 was 1.05, which indicates the presence of anxiety symptoms. T1 mean score was 0.42, and T2 mean score was 0.54, highlighting a complete resolution of the symptomatology (ΔT1-T0: .007; ΔT2-T0: p = 0.001).

Insomnia was evaluated using the Insomnia Severity Index (ISI): despite a global improvement in the mean scores before- and after-treatment (T0: 7.43; T1: 4.53; T2: 2.25), this did not reach statistical significance (see **Table 3**).

**Table 3:** Changes in psychopathological symptoms in subjects with Cocaine Use Disorder during rTMS treatment.

	T0	T1	T2	Z T1-T0	Z T2-T0	Sig. T1-T0	Sig. T2-T0
**Global Severity Index (SCL-90)**	0.98 ± 0.74	0.47 ± 0.29	0.47 ± 0.44	−2.715	−2.689	**0.007**	**0.007**
**Depressive symptoms**							
BDI scores	17.19 ± 12.40	7.25 ± 6.54	7.38 ± 6.74	−3.018	−2.672	**0.003**	**0.008**
SCL-90 Depression Scale	1.24 ± 0.94	0.54 ± 0.45	0.67 ± 0.59	−2.867	−2.989	**0.004**	**0.003**
**Anxiety symptoms**							
SAS scores	36.37 ± 10.34	33.20 ± 8.92	28 ± 4.53	−0.825	−3.183	0.410	**0.001**
SCL-90 Anxiety Scale	1.05 ± 0.77	0.42 ± 0.37	0.54 ± 0.46	−2.708	−3.197	**0.007**	**0.001**
**Insomnia**							
Insomnia Severity Index	7.43 ± 6.51	4.53 ± 3.7	2.25 ± 2.86	−1.301	−1.768	0.193	0.077

Bolded text: p < 0.05

**Figure 1:**
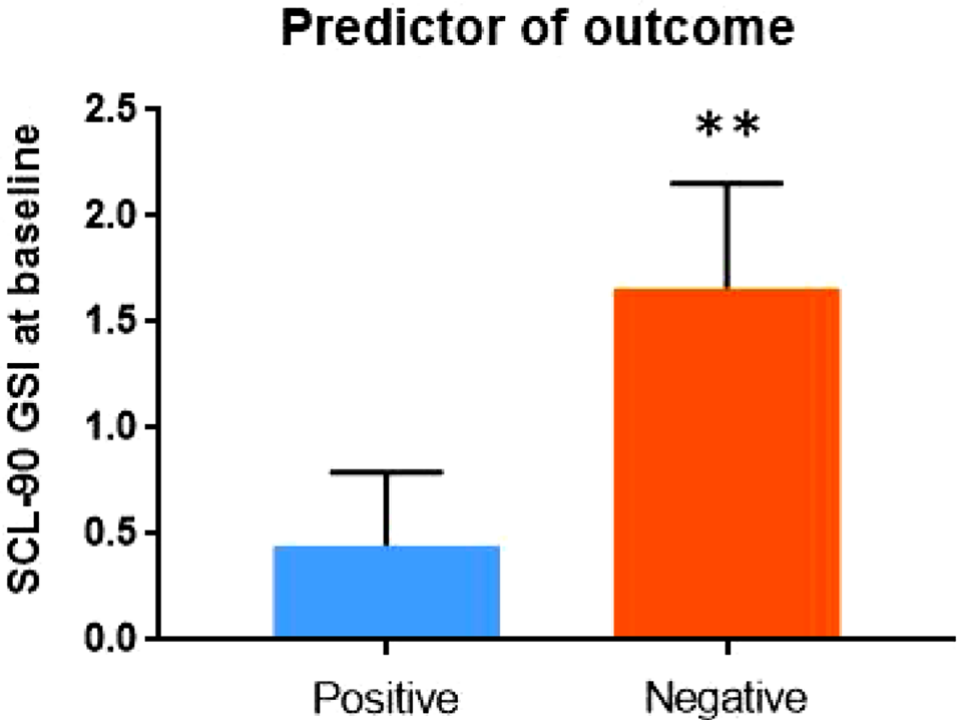
Psychopathology at baseline – as measured by the Global Severity Index (GSI) of Symptom Checklist-90 (SCL-90) – may predict outcome of rTMS treatment.

Finally, in order to explore the potential impact of baseline psychopathology on clinical outcome, we included in the positive outcome group all subjects who tested negative at the urinalysis at the end of the study (N = 9), and in the negative outcome group all patients who relapsed in cocaine use after rTMS treatment (N = 7). No impact of current pharmacological treatment was detected on treatment outcome. In terms of overall psychopathological burden, subjects who had baseline lower scores at SCL-90 scale were more likely to be in the positive outcome group at the end of the study (Z = -3.334; p = 0.001) (**Figure 1**).

## Discussion and Conclusions

In this study, we confirmed the efficacy of high-frequency rTMS of the DLPF in Cocaine Use Disorder, showing a peculiar effect on specific psychiatric symptoms that may, to some extent, contribute to its anti-craving and relapse preventing properties.

In our study, a protocol characterized by an intensive stimulation treatment of twenty sessions over two weeks was proposed for the first time, showing a differentiating aspect with respect to other studies, in which a lower number of intensive sessions was preferred (24, 25, 34, 35). Our protocol was designed on the basis of recent findings in the area of depressive disorders, where a higher number of rTMS sessions was positively linked to rTMS effectiveness in reducing depressive symptom severity (36).

In comparison to other studies on CUD (24, 25, 34, 35), our sample is in line with gender ratio, with a higher prevalence of males. This gender difference is consistent with the epidemiology of cocaine consumption in Italy, according to the Drug Report for our country recently published by the EMCDDA (37). In fact, 92% of the clients accessing treatment services for cocaine use disorder in 2015 were men, with a majority of cocaine users snorting cocaine.

In this open sample, rTMS appears to elicit its more notable effects on depressive and anxiety symptoms, confirming previous data from our group, according to which the pro-hedonic effect of rTMS is crucial and directly related to the reduction of cocaine craving (27). On the other hand, the improvement of depressive symptoms was not evidenced in similar studies (24, 35), whereas other studies, which used deep TMS and were limited by the low number of participants, did not evaluate these aspects (25, 34). As a whole, it should be taken into account that CUD and depression have a high comorbidity (38), and a reduction of cocaine craving may also be an indirect outcome of rTMS *via* an improvement of depressive symptoms. Multiple findings suggest that symptoms pertaining to the area of depression may predict drug use outcomes: the presence of depressive symptoms worsens cocaine use outcomes in patients treated with a 12-step focused group continuing care (39), and depression severity significantly influences future increase in drug use (40). Moreover, patients who score higher in depressive scales are more likely to fail abstinence after treatment in outpatient substance misuse services (41). On the other hand, we should consider that in cocaine use disorders pharmacological treatments based on antidepressants did not evidence significant data in terms of craving reduction and relapse prevention (42, 43). Therefore, the hypothesis of an “indirect” effects of rTMS on craving *via* an improvement of depressive symptoms should be taken into account with caution, hypothesizing for rTMS an adjunctive independent effect on different pathways, with frequent overlaps and cumulative effects. However, in order to clarify these aspects, further larger trials are needed.

Another hypothesis that may explain our results follows the theories by Hanlon and colleagues (44, 45). As reported in their recent studies, two neurobehavioral systems may be targeted by TMS in order to treat cocaine use disorders: an executive control system, namely the dorsal lateral frontal–striatal, likely involved in resisting drug use, and an impulsive system, namely the ventral medial frontal–striatal, likely involved in drug-associated craving and use. It may therefore be useful to either increase activity in the DLPFC-dorsal striatal circuit, as in most of the previous trials in cocaine use disorders and in the present study, or to decrease the activity in the ventral medial prefrontal cortex–caudate circuit using an inhibitory rTMS (1 Hz or cTBS) (45). It is therefore a possibility that the stimulation of the DLPFC may be less associated with a direct anti-craving effect, probably exerting its action in terms of relapse prevention partially through other mechanisms, as evidenced in this paper. In this regard, it is also of some interest that subjects exhibiting baseline lower scores of psychiatric burden were more likely to be in the positive outcome group at the end of the study, confirming a specific role of rTMS in Cocaine Use Disorder, regardless of its direct effect on other psychopathological dimensions. However, it should be also considered as a limitation of our study that craving was evaluated with an unspecific scale (CSSA), and that the small sample size and the lack of neuronavigation could have tempered the magnitude of the effect.

Consistently with previous pilot evaluations by our group (27, 46), we observed an improvement in the hedonic tone of the subjects. As previously mentioned, anhedonia is a condition in which a subject shows a lost or diminished ability of experiencing pleasure; it represents one of the two main diagnostic features of depressive disorders. This symptom was significantly reduced between T0 and T2. Anhedonia is believed to have a role in relapsing, and it may also be involved in transitioning from a recreational substance misuse to an actual substance use disorder. Moreover, in addicted patients, anhedonia has been positively correlated with craving, intensity of withdrawal symptoms, and some temperamental factors (38, 47). Anhedonia appears to have a peculiar association with stimulant use: in CUD patients, the anhedonia-cocaine relationship remains significant after adjusting for demographic, psychiatric, and non-stimulant substance use (48). This has also been evidenced in animal models; in fact, cocaine-sensitized rats show anhedonia-like behaviors, which may be reversed by the administration of imipramine (49).

Anxiety appeared to be not a primarily relevant symptom in our sample, as only few (17.6%) of the patients scored for mild/moderate anxiety at the Zung Self-Rating Anxiety Scale; on the other hand, however, mean scores for the anxiety subscale of the SCL-90 revealed the presence of clinically relevant anxiety symptoms. Anxiety may represent a core clinical feature of cocaine withdrawal, possibly related to the hyper-activation of brain stress systems mediated by corticotropin-releasing factor, noradrenaline and dynorphin (50, 51), combined with the inactivation of Neuropeptide Y (NPY) and the brain anti-stress system (50), and other hormones and modulators dysregulation (52). Anxiety sensitivity is considered to be a prospective predictor of treatment dropout in crack/cocaine users: individuals with higher anxiety are more likely not to succeed in completing detoxification treatments (53). A reduction in anxiety scores may therefore predict a positive outcome with respect to cocaine use relapse. To this respect, insomnia may represent a relevant issue in terms of cocaine relapse, too. However, in this study we did not evidence a significant effect of rTMS on this dimension but only a general trend. This is probably due to the small sample size and the presence of different confounders (depressive symptoms, anxiety, anhedonia).

This study has several limitations: 1) the open design and the limited number of participants narrow the interpretation of results; 2) the follow-up period is limited, and this does not allow to draw long-term prevision for a chronic relapsing disorder as cocaine addiction; 3) rTMS stimulation of the DLPF was not neuro-navigated; although the standard evaluation of the area pertaining the DLPFC has shown a good level of reliability (30), the absence of a precise methodology does not consent a rigorous evaluation of the area, which is mostly associated with therapeutic improvements, as recently showed (54), and could represent a limiting factor to the potentiality of this protocol in terms of craving reduction; 4) the lack of a distance-adjusted motor threshold may mean that patients were under-stimulated and that they did not receive the same TMS dose. Interestingly, this limitation may explain, to some extent, the high number of non-responders in our sample. It is well known that the distance between the scalp and the cortex may influence TMS effectiveness. In fact, the skull and scalp may influence the impedance and, consequently, the TMS-induced electric current within the cerebral cortex (55). In this context, several shreds of evidence suggest that the resting motor threshold (which depends on the motor cortex depth) should be adjusted for the differences in depth between nonmotor cortical regions and the motor cortex (M1) (55). In our feasibility study, subjects did not undergo MRI. The lack of structural imaging data has prevented us from adjusting the stimulation intensity based on the distance between scalp and cortex at the target and this may account for differences in TMS dose between subjects and, even more critical, an insufficient stimulation of the DLPFC. Cocaine users usually exhibit significantly higher RMTs than healthy controls (56, 57). Even if the effects of cocaine on cortical inhibitory and excitatory circuit has not been well explained, cocaine abuse has been proved to increase cortical excitability (increased intracortical facilitation) (56, 57) and it has been suggested that the increased RMT is an adaptation mechanism to this increased excitability (56). Moreover, Hanlon et al. showed that RMTs do not respect the correlation with brain-scalp distance among cocaine users (57). Given the lack of scalp–brain distance adjusted RMT and in order to reduce the risk of adverse effects such as seizures (58), we decided to set stimulation intensity to 100% of RMT, lower than the 120% RMT intensity suggested by depression protocols.

In conclusion, this study is consistent with previous contributions concerning the use of rTMS in subjects with cocaine use disorders, showing a lower but significant effect on craving for the whole sample, and a more specific action in other psychopathological areas able to exert an indirect effect in terms of relapse prevention. Our results also allow to speculate that TMS on the DLPC may be more effective in selected subgroups of addicted patients, namely those with concurrent mild depressive symptoms and, more probably, a relief-type craving (59). In the next few years, TMS should undergo scrupulous evaluations through hypothesis-driven research, in order to proceed with its validation as a therapeutic option for addictive disorders (60). A double-blind, sham-controlled, neuro-navigated rTMS study design is mostly needed, in order to confirm the potential benefits of this technique, opening new scenarios in substance use disorders treatment.

## rTMS Stimulation Group Collaborators

Luisa De Risio, Ilaria Petrucci, Gaia Tourjansky, Patrizia Capicotto, Francesca Neri, Gianluca Ruggiero, Barbara Cassiani, Silvia Fraticelli, Valentina Moroni.

## Ethics Statement

The study was approved by the Department Review Board and by the “University of Chieti” Ethic Committee. All subjects gave written informed consent in accordance with the Declaration of Helsinki.

## Author Contributions

GM, MP and MdG designed the study protocol. The *rTMS stimulation group* assessed the patients and delivered neuromodulation protocol. MP and RS performed the statistical analysis: RS and CM wrote the introduction. MP wrote the *Materials and Methods*, and *Results* sections. GM, MP, CM and RS wrote the discussion. MG and FF reviewed the discussion.

## Funding

This work was supported by the “Departments of Excellence 2018-2022” initiative of the Italian Ministry of Education, University and Research for the Department of Neuroscience, Imaging and Clinical Sciences (DNISC) of the University of Chieti-Pescara.

## Conflict of Interest Statement

The authors declare that the research was conducted in the absence of any commercial or financial relationships that could be construed as a potential conflict of interest.
